# Health care stakeholder perspectives regarding the role of a patient navigator during transition to adult care

**DOI:** 10.1186/s12913-019-4227-6

**Published:** 2019-06-17

**Authors:** Gina Dimitropoulos, Elizabeth Morgan-Maver, Brooke Allemang, Kyleigh Schraeder, Shannon D. Scott, Jorge Pinzon, Gail Andrew, Gregory Guilcher, Lorraine Hamiwka, Eddy Lang, Kerry McBrien, Alberto Nettel-Aguirre, Daniele Pacaud, Lonnie Zwaigenbaum, Andrew Mackie, Susan Samuel

**Affiliations:** 1 Faculty of Social Work, Professional Faculties 4212, University of Calgary, 2500 University Dr. NW, Calgary, AB T2N 1N4 Canada; 2 Department of Psychiatry, University of Calgary, Calgary, Alberta Canada; 3 Alberta Children’s Hospital Research Institute, Calgary, Alberta Canada; 4 University of Calgary, Calgary, Alberta Canada; 5 Department of Pediatrics, University of Calgary, Calgary, Alberta Canada; 6 Faculty of Nursing, University of Alberta, Edmonton, Alberta Canada; 7 Department of Pediatrics, University of Alberta, Edmonton, Alberta Canada; 8 Section of Pediatric Oncology and Blood and Marrow Transplant, Alberta Children’s Hospital, Calgary, Alberta Canada; 9 Section of Nephrology, Alberta Children’s Hospital, Calgary, Alberta Canada; 10 Department of Emergency Medicine, University of Calgary, Calgary, Alberta Canada; 11 Department of Community Health Sciences, University of Calgary, Calgary, Alberta Canada; 12 Department of Family Medicine, University of Calgary, Calgary, Alberta Canada; 13 Section of Diabetes and Endocrinology, Alberta Children’s Hospital, Calgary, Alberta Canada; 14 Division of Cardiology, Stollery Children’s Hospital, Edmonton, Alberta Canada

**Keywords:** Adolescents, Young adults, Transition age young people, Chronic disease, Qualitative research, Patient navigator (PN), Special health care needs (SHCN)

## Abstract

**Background:**

Transition to adult care represents a vulnerable period for young people with special health care needs as they navigate multiple life transitions and developmental issues. Patient navigators are a promising intervention designed to facilitate the transfer from pediatric to adult care. However, consistent definitions, key tasks, roles and responsibilities are lacking in guiding the scope of practice and the implementation of patient navigators.

**Methods:**

Fundamental qualitative description was utilized in this study to identify perceptions from health care providers about implementing a patient navigator service for young people with special health care needs in transition to adult care. A purposive sample of health care providers with a variety of backgrounds within pediatric and adult systems in Alberta, Canada were recruited. Semi-structured interviews with participants were analyzed using thematic analysis to inductively identify perceptions regarding the role of patient navigators.

**Results:**

A total of 43 health care providers highlighted the need for a patient navigator service to encompass 4 key stages for young people with special health care needs transitioning from pediatric to adult services: (1) identification of young people with special health care needs and families requiring support, (2) preparation for transfer, (3) health system navigation and, (4) post-transfer support.

**Conclusions:**

The results of this qualitative study provide guidance for the development of patient navigator interventions for young people with special health care needs, as well as provide support for current transition services offered across Canada.

**Electronic supplementary material:**

The online version of this article (10.1186/s12913-019-4227-6) contains supplementary material, which is available to authorized users.

## Background

Young people with complex or special health care needs (SHCN), are defined as those who acquire, or are at increased risk for, a chronic physical, mental health and/or developmental (including cognitive and sensory impairment) condition(s) [1, 2]. These young people often require transfer to adult health care services. Currently, the post-transfer period is associated with significant loss to follow up in medical care. Many young people and their families are unprepared for the transfer and face challenges navigating adult health care services [2–4]. Sub-optimal transfer to adult care may lead to increased health care utilization and patient’s deterioration in health [5–10]. A coordinated and planned health service transfer to adult care is necessary to ensure optimal outcomes [2].

Various interventions have been recommended to support young people with SHCN during their transition from pediatric to adult oriented health care services [11, 12]. The Canadian Association for Paediatric Health Centres in its Guideline for Transition from Paediatric to Adult Care recommends that health care organizations implement transition planning by care coordinators/navigators [2]. In the literature, the role of facilitating transition of care has been ascribed to individuals with varied roles, including: transition care coordinators [13], patient navigators [9], community health workers [14], and case managers [15]. However, these terms are used interchangeably in the literature and distinct definitions of each role are poorly articulated [11].

Patient navigators (PNs) have been used in populations with a variety of health conditions [13, 15–17], primarily in adult settings, to support care coordination and sustained engagement with health services [18]. Care coordination and transition intervention models have been piloted to support children with medical complexity and their families utilizing pediatric nurses acting as case managers and transition educators [19–21]. The interventions described in these studies focused on education initiatives, promoting self-management, resource navigation and assisting with appointment scheduling with providers. Results showed that these models reduced hospitalization costs, family out-of-pocket costs, length of stay in hospital, improved patient satisfaction, reduced likelihood of delays accessing adult care and improved knowledge and self-management skills [19, 20, 22].

Intervention studies demonstrate that the use of coordinators improves appointment attendance, medication adherence and reduces loss to follow-up in specific patient populations. A recent Canadian environmental scan of existing navigation services for patients 0–19 years identified 23 pediatric PN programs across the entire country [23]. Despite the emerging use of PNs, there remains a lack of consensus on their role, responsibilities, and qualifications in the context of supporting young people with SHCN navigating transition to adult care [24, 25]. The purpose of this paper is to describe health care providers’ perceptions about the PN role in supporting young people (including adolescents and young adults aged 13 to 24 years old) with SHCN during transition to adult care including scope, responsibilities and tasks. Key stakeholders are defined as health care providers, policy makers and administrators in the pediatric and adult systems in Alberta with expertise in health care transition.

## Methods

This study adheres to the COnsolidated criteria for REporting Qualitative research (COREQ). The researchers in this study employed fundamental qualitative description with the goal of describing rather than interpreting data. Fundamental description is commonly employed to garner information from applied health care contexts that may be used to inform service and practice-based changes [21, 22]. We intentionally employed this approach to glean what practitioners are employing in practice that may be beneficial to young people in the health care system.

### Population and setting

Participants were recruited from primary, tertiary and community-based health settings in the province of Alberta, Canada. A total of 37 individuals expressed interest in this qualitative study and were screened for participation. Three potential participants could not be scheduled for an interview within the specified recruitment period, therefore, 34 key stakeholders were included in the final sample. Participants’ primary work locations were based in two different city centres (Calgary (65%) and Edmonton (27%)) as well as semi-urban/rural areas (9%). The majority of participants (Table 1) were female (79%), between 40 and 60 years of age (82.4%) and worked with adolescents (59%), both adolescents and adults (21%) or adults only (18%). Occupations of the participants included administrators/policy makers (*n* = 13), direct service providers (*n* = 27) and clinicians primarily conducting research in their clinical setting (*n* = 2). Those who identified as researchers in this study were also clinicians involved in direct service provision to young people with chronic conditions. Five transition coordinators were interviewed for this study who primarily worked in the pediatric sector.

**Table 1:** Characteristics of key stakeholders participating in focus groups and interviews

Characteristic	No. (%) of participants *n = 34*
Gender	
Female	27 (79%)
Age	
< 40	3 (9%)
> 40	28 (82%)
Work location	
Calgary city	22 (65%)
Edmonton city	9 (27%)
Semi-urban/rural Alberta	3 (9%)
Participant roles	
Policy maker/Administrator	13 (38%)
Family advisor	1 (3%)
Physician	5 (15%)
Nurse	8 (24%)
Social Worker	4 (12%)
Dietician	4 (12%)
Researcher	2 (6%)
Transition Coordinator	5 (15%)
Years of experience in role	
Less than 5	5 (15%)
5 to 10	8 (24%)
11 to 19	7 (21%)
More than 20	14 (41%)
Identified as being in leadership position	9 (27%)
Working with primarily	
Adolescents only	20 (59%)
Adults only	6 (18%)
Adolescents and adults	7 (21%)
Work setting	
Primary care (adolescents)	7 (21%)
Primary care (adults)	2 (5%)
Tertiary care (adolescents)	19 (56%)
Tertiary care (adults)	6 (18%)
Community-based setting	6 (18%)

### Sampling and recruitment

We used purposive sampling [26] to identify key stakeholders connected to service delivery of pediatric and adult health care within Alberta Health Services, the health authority responsible for the majority of health service delivery for Albertans. Key stakeholders referred to policy makers, administrators, researchers, or clinicians who work in pediatric and/or adult systems. At the start of study, two authors (GD, SS) presented the research project to a provincial network focused on transition issues for young people in Alberta [27] and recruitment material was distributed to attendees (including clinicians, policy makers, and researchers) through email. Interested stakeholders contacted researchers expressing their interest to participate in the study. A member of the research team reviewed the study purpose, data collection methods and data storage procedures. They were informed that all transcripts would be de-identified in order to protect and maintain the confidentiality of all participants. Participants then signed informed consent forms. Participants were offered the opportunity to participate in a focus group during specific dates and times. If they were unable to participate in the scheduled focus group, they were offered a one-one-one interview at their preferred date and time. The interviews were offered by telephone or in person at the participants’ convenience. The same interview guide was used in both focus groups and one-on-one interviews and no differences emerged in the demographics including professional backgrounds of participants who were involved in the interviews versus focus groups, as demonstrated by the diversity of participants represented in the focus groups.

### Inclusion and exclusion criteria

Inclusion criteria for participation were: a) provide direct or indirect services to individuals between ages of 13 to 24 with a chronic health condition and SHCN in Alberta; and, b) the provision of services to support transfer of care from pediatric to adult services. We excluded practitioners who cared for patients with mental health and neurodevelopmental disorders solely, as these individuals typically work outside tertiary care centres in community-based organizations. A brief screen for inclusion was facilitated by a team member.

### Data collection procedures

A semi-structured interview guide was developed by the author (GD) with input from co-authors and local experts in transition. Their backgrounds included a diverse expertise in social work, community health sciences, adolescent mental health, and youth transition to adult care. The guide was piloted with two pediatric health care providers. This guide was refined based on the pilot interviews and used for individual interviews and focus groups (Additional file 1). All focus groups were conducted by one of the authors (GD or EM) within one pediatric tertiary care hospital (Alberta Children’s Hospital), and were approximately 90 min. Interviews were conducted by two authors (GD, EM) in-person or by telephone, lasting 40–60 min.

### Analysis

Qualitative interviews and focus groups were audio-recorded and transcribed verbatim. Data analysis was conducted concurrently. The two independent analysts paid close attention to the descriptions that participants in the individual interviews and focus groups provided to questions about the role of the patient navigator. Upon review of all transcripts, it appeared that no significant differences were uncovered in participant responses between the interviews and focus groups, therefore, the transcriptions from both methods of data collection were analyzed simultaneously. In our study, combining focus group and interview data enhanced the trustworthiness of our findings given the convergence of central themes amongst the participants [28]. The team discontinued recruiting participants due to the strong quality of the dialogue when it was agreed that the information obtained achieved sufficient power to fulfill our study aim [29].

The team used thematic analysis and systematically adhered to the six steps described by Braun and Clark [30]. We employed an inductive approach given the paucity of literature on health care providers’ perceptions of the patient navigator in supporting YSHCN to transition to adult services. First, two coders (EM, BA) immersed themselves in the data by reading and re-reading transcripts in order to familiarize themselves with the data. Second, using memos, the team documented their impressions of the data and independently generated and assigned codes to the qualitative data. Codes refer to words that describe the researchers’ thoughts about ideas that emerged within and across the data [31]. Third, the data were collated based on codes initially established. The team debriefed about the codes generated by each coder and only codes that received consensus were included. Codes were grouped into major themes and given titles, in order to better represent the data. Fourth, emergent themes were reviewed and refined to ensure they were representative of the data. Next, the themes were defined, named and further refined while identifying subthemes where applicable. Finally, for the sixth step, the research team analyzed and described the themes and subthemes and created a visual concept map [32].

Several steps were undertaken to maximize methodological rigour by following the guidelines for publication of qualitative research [32]. First, we worked with experts in transitions and chronic illness to develop an interview guide. We used thick descriptions in our results section by integrating direct quotes from participants to illustrate the concepts and themes generated [33]. In qualitative research thick description refers to the utilization of quotes that provide a visual description or depiction of the themes. Throughout the data analysis process, the principal investigator facilitated weekly peer debriefing with the research team (two qualitative research assistants) to provide a forum for reflection of the codes that were emerging from the data. Member checking was used to obtain feedback from others in similar roles. Finally, a concept map was created to provide underlying structure of the data, and exemplar quotes are provided to illustrate the verbal narrative of themes. Transcripts were not returned to the participants for comment and participant checking was not performed.

## Results

### Final sample

Twenty-four individual interviews, and 3 focus groups of 2, 3 and 5 participants each were conducted between July and November 2017. Although focus groups were small, our research team facilitated discussions between participants regardless of the size and encouraged varying opinions to each other’s responses to the same questions.

### Findings

**Fig. 1:**
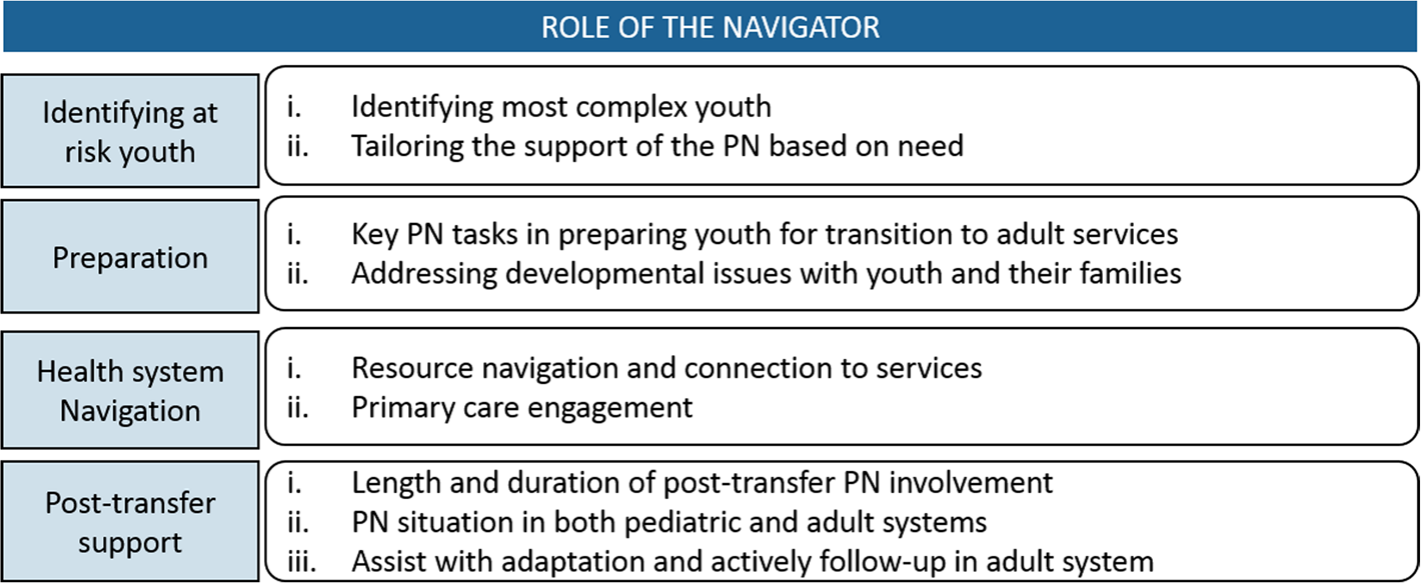
Conceptual Map- stages defining the role of the patient navigator

The overarching theme emerging from the data was a perceived need for PNs to be involved at multiple stages of the transition experience. Participants identified four stages of PN involvement: [1] identification of young people with SHCN and families requiring support, [2] preparation for transfer; [3] health system navigation [4] post-transfer support. Figure 1 provides a visual depiction of the stages.

Themes and sub-themes from the analysis are described with exemplar quotes below. Quotes are referenced by occupation and an identification number.

### Identification of young people at risk

Participants shared that that a PN should not be universally available to every young person who is transitioning to adult care. “*I don’t think all kids need it [navigator]*” (nurse, participant 02), providing services to those who need it the most was a common theme across participants. However, it is important to *“come up with some criteria of who are we really looking for, who is at risk of having a poor transition”* (nurse practitioner, participant 08).

#### Identifying the most complex young people

Participants recommended screening young people and families for complex needs, *“kids who have multiple diagnoses,”* (nurse practitioner, participant 08) *and “mental health comorbidities,”* (physician, participant 14), whom they believed greatly needed additional emotional and instrumental support. Participants also commented that a PN should provide support to young people with developmental disabilities, precarious housing, poverty, mental health problems, substance abuse, and limited natural supports. “*Clear red flags- so I think if you’re in the judicial system, if you’re in the foster system, if you have no place to live, the street, those are general but I think they are applicable. If you have a special need, I don’t know how you want to take care of your basic needs …*” *(*physician, participant 14*).*

All participants reported that young people and families who are unfamiliar with the Canadian health care system would benefit from the service. As one participant stated: *“families …*. *who haven’t grown up here. English as a second language, maybe recently immigrated to Canada, have no social supports, they are struggling on lots of different socioeconomic levels”* (nurse, participant 01). Further, young people *“thrown into this world of a new diagnosis and you don’t have a clue [of] the things you need,”* (nurse, participant 01) at the time of transfer were also perceived to need support from a PN. Participants also identified a critical need to support young people who frequently use the emergency department. “*And potentially if I know what the vision is, if there are flags that this person is at risk, if there isn’t some oversight and they are showing up in emerg or having admissions, how can they potentially support what needs to happen in a bit more of a proactive way*” *(*administrator, participant 09).

#### Tailoring the support of the PN based on need

Participants endorsed tailoring support to each patient and their unique circumstances once a PN is employed. The support could vary from providing educational resources to the provision of more intensive and prolonged support to increase engagement in adult programs for young people who are more complex and disadvantaged*.* One participant described this idea using the analogy of traffic signs: *“If we look at green, yellow and red flags. The green flags are the families who are managing care really well, attending all appointments - they are the ones who maybe need some paper resources and figure out early on where do I go for my care when I’m an adult. If we look at the yellow flags, those are … the kids who have multiple diagnoses [and] would be more at a risk because they are going to have to find multiple care teams. And the red flags are the kids who are falling through the cracks already who have higher risk of death or disability if they transition.”* (nurse practitioner, participant 08). The diversity among PN tasks was summarized as: *“teaching, follow up, ensuring supports are in place if needed, answering questions, providing educational material, teaching other staff how to implement some of the basic teachings so it doesn’t all fall on one person, incorporating other key members as needed …*. *And just coordinating everything.”* (nurse, participant 25).

### Preparing for transition

Participants identified two key tasks of a PN prior to transition; 1) preparing young people and families for transition to adult services and 2) addressing developmental issues with young people and their families to support a successful transition.

#### Key tasks during preparation

**Fig. 2:**
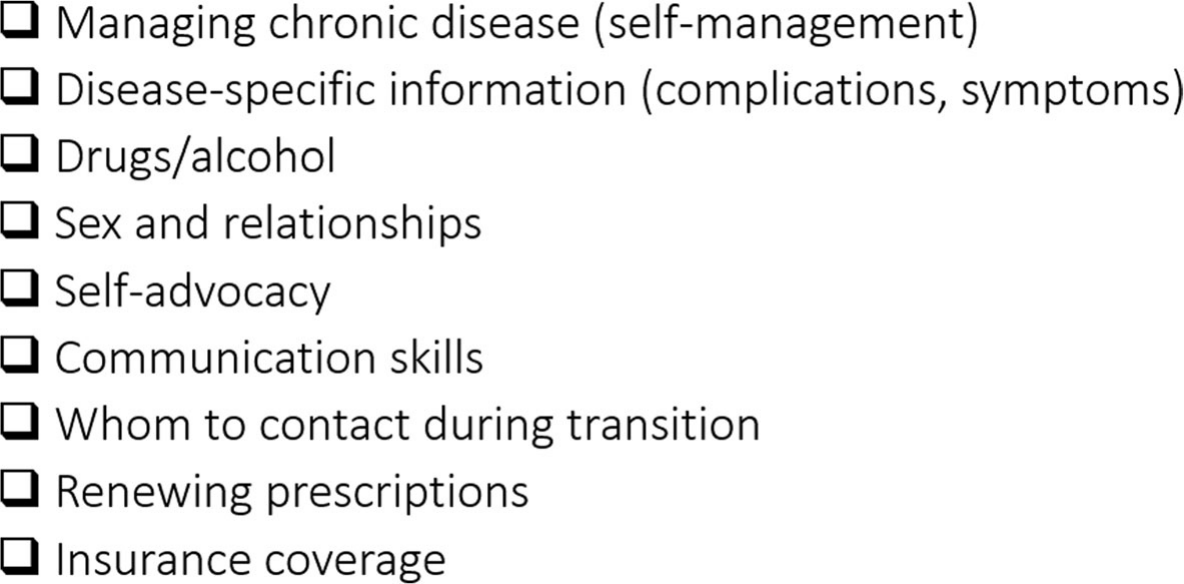
Transition preparation education topics

Participants agreed the PN should carry out transition readiness assessments, and use these assessments to guide the support they provide during the preparation for transitioning to adult care. Common suggestions were that the PNs help young people build their self-advocacy, self-management and communication skills to prepare for engaging with adult providers. As summarized by a nurse (participant 25): *“ideally, [young people] go into the adult world with complete knowledge … what possible complications that they could have, are able to advocate for themselves, talk to medical professionals, be able to articulate what they need in a clear manner”.* Several participants articulated the PN act as an adjunct to the clinical team and provide necessary education to the young people about their health condition when knowledge gaps exist, *“give them the knowledge and empower them to be more aware of what is going on as opposed to the shock of walking into appointments”* (nurse, participant 25) and coach them to effectively communicate their needs to health care providers. PNs can play a role in *“making them comfortable with the idea that they are going to be seeing new providers and that they want to help you … giving them more information about how to ask questions.”* (nurse practitioner/transition coordinator, participant 15). Participants also advocated that a PN provide education in broad topics (Fig. 2) to prepare for the transition to adult care.

#### Addressing developmental issues with young people and families

There was consensus among participants that transition preparation should begin early but this ranged from age 12 to 16. As a nurse (participant 25) stated: “*hopefully eventually in the 14-16 year old range, and working slowly to transition them and help them with that process getting into that adult world.”* Participants also advocated for a lifespan perspective that facilitates the young person achieving their goals with greater independence. Participants agreed that PNs should work with both young people and parents, but aims shift based on developmental needs, and degree of functioning due to illness and cognitive abilities. One participant proposed: “*that it’s really dependent on how functional the parents are and what connection they have with their child, and how functional the young adult is and what degree of development. There’s enough variability there to have a check-in with clinical staff who work with them and should have a pretty good knowledge of them”* (physician/researcher, participant 07). Key responsibilities for preparing young people for transition, including promoting self-management and self-advocacy skill building, depend on the needs and developmental stage of the young people. One participants shared: “*For chronic disease management, it’s a continuing process. Because transition is through our whole life. What a kid is able to do at age 12 versus 14 versus 16 is completely different. And what you’re trying to do is balance what they are able to do … and have parents support the rest”* (nurse/transition coordinator, participant 15).

### Health system navigation

One of the most common themes identified by participants was the role of the PN in health system and resource navigation and facilitating engagement with primary care.

#### Resource navigation and connection to services

**Fig. 3:**
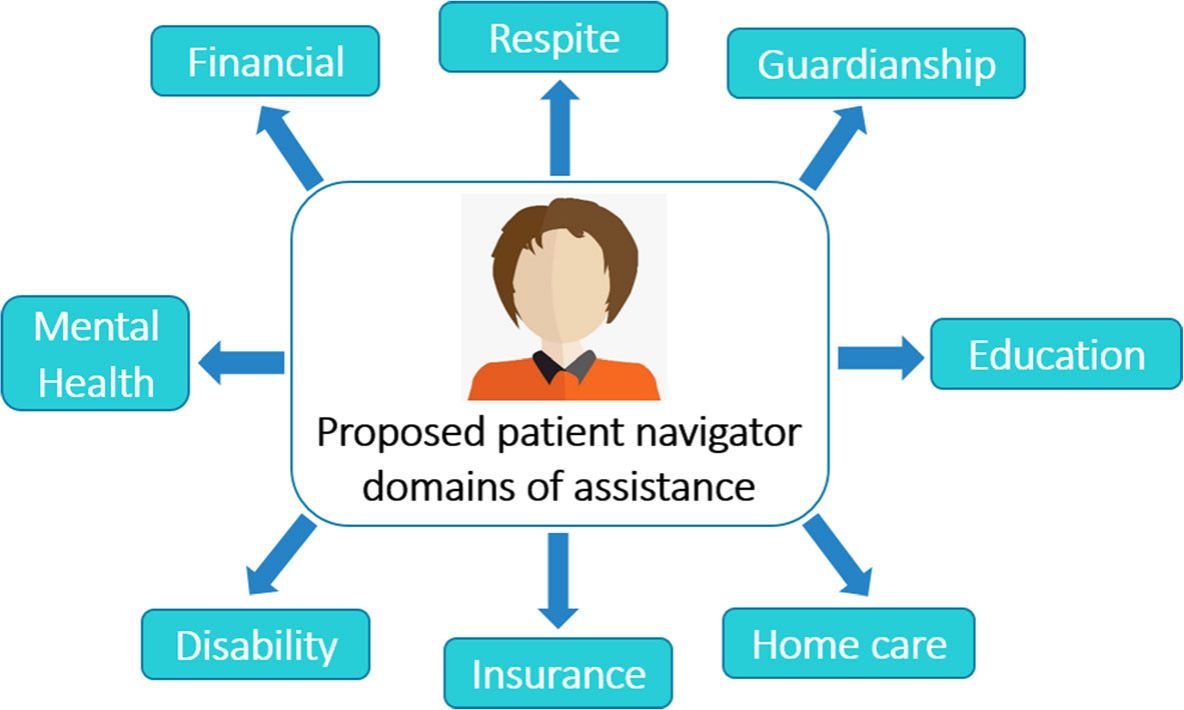
Proposed domains patient navigators can assist with access

Participants described the PN’s main involvement as *“reaching out to certain systems or at least connecting families,”* (nurse, participant 01)*,* thus connecting young people to diverse multi-disciplinary services in the community, as summarized in Fig. 3. The PN represents *“a bridge”* (administrator, participant 09), “*a person … kind of in the middle”* (nurse, participant 01*)*, between pediatric and adult care providers who are often dispersed rather than in one location. One participant (administrator, participant 11) said *“it’s sort of like you think about a grassy yard on a corner where there is a sidewalk that turns 90 degrees… I think about those navigators as those people who cross the corners and start to beat down a path.”*

#### Primary care engagement

Participants consistently emphasized that primary care providers ensure a seamless transition by providing continuity of care. As expressed by one participant (administrator, participant 20), *“every single one of these transition patients and families should have a primary care physician regardless of how complex they are.”* Another participant stated, “*there’s no question that the family doctor is part of the team and they are the anchor for that family.”* (nurse/transition coordinator, participant 15). Additionally, participants perceived that PNs “*should make sure that the patient they are navigating can be settled well in a primary care service*” (policy maker, participant 05). Another participant also mentioned the importance of a “*medical home*” (nurse/transition coordinator, participant 15*)*, as a part of better health care for young people who leave pediatric services. Many also advocated for increased support for primary providers in order to improve their understanding of the unique developmental characteristics of young people with special health care needs. As advocated for by an administrator (participant 09): “*Another big piece working with adult partners is helping them understand what are the needs of adolescents versus other adults and seniors*”.

### Post-transfer support

Support following transfer was considered critical to promote continued engagement and assist with “*attachment and adaptation*” (administrator, participant 09) of transitioning young people to adult health systems.

#### Duration of post transfer involvement

Opinions regarding length and duration of post-transfer PN involvement varied. Some participants expressed views that the PNs’ work should not be limited by time and continue until all patient needs have been addressed due to the immense need for continued support in adult health services. An administrator (participant 24) explained, “*I think it comes down to resources but I think they should always be involved as long as somebody continues to transition through the system*”.

Others argued for a prescribed period of intervention; *“I would say 2 years after transfer of care. I think we shut it off far too quickly. And the kids that are falling through the gaps, it would be great if they have somebody that they can call … that contact of trying to scrape those kids off the floor and get them back into health care would really help.”* (nurse/transition coordinator, participant 15).

#### PN situation in both pediatric and adult systems

All participants agreed that PNs should have office space in both pediatric and adult systems as illustrated by this quote: “*I think both. I think the navigator should be able to cross the boundary. It’s not really a [pediatric] service or adult service. If we’re staying in the true sense of a navigator, like providing one on one care coordination it will depend on where that young people is having problems … I guess they should be able to cut across*.” (policy maker, participant 05). Many participants expressed a greater need for the PN to work within adult-focused programs, which often lack the resources to implement similar levels of support to patients and families.

#### Assist with adaptation and actively follow-up in the adult system

Across all interviews participants discussed the need for the PNs to assist with adaptation to the adult system and conduct follow-up so *“that patients are not lost to care”* (dietician, participant 03). One participant proposed a PN “*would encourage, she would follow up on appointments and making sure people got attached. She would send reminders about appointments, and if people missed them she would encourage them to reschedule”* (dietician, participant 03). Some participants stressed the need for a PN to follow up with patients and intervene as necessary to help manage adverse events. As stated by one participant: “*it needs to be able to come in and support if things aren’t going well, or we can anticipate there being challenges with this child or family*” (administrator, participant 09). Many participants added there is a greater need for the PN to facilitate attachment to adult-focused programs since adult providers often lack the time to engage young adults due to limited time and resources.

## Discussion

Participants provided rich and predominantly consistent perspectives regarding the role of a PN to support young people with SHCN transitioning to adult care. In our study, participants agreed that “patient navigator” is a term that closely captures and encompasseses the variety of roles that are undertaken by professionals working with young people with SHCN. There was greater variability among participant opinions regarding the age at which transition preparation should begin and the duration of patient navigator support. Screening for case complexity based on medical and mental health comorbidity, transition readiness, and family context were identified as important steps in determining who will have access to the PN. Empowering young people with knowledge and supporting them to take responsibility for their care over time arose across the interviews and focus groups. Provision of developmentally appropriate supports, health system navigation and post-transfer assistance were also identified as important themes.

The existing literature broadly aligns with our findings of providing support to populations at risk of poor outcomes [34]; those with complex medical or mental health comorbidity [35], with low health literacy [36], new immigrants [37] and those within complex social situations [38]. Our findings about the patient navigator’s role in preparing young people for transition to adult services are similar to those summarized by Luke et al. [23], where existing Canadian programs are described as aiming to promote care coordination, education and emotional support. Many of the proposed key tasks of a PN in the context of transition preparation (education, transition resource development), also align with previously published transition support programs that achieved favorable system and patient level outcomes [9, 13, 15]. Studies from the patient perspective indicate that adolescents value trusting relationships with health care providers who offer adolescent-focused information and discuss aspects of their lives outside of the illness [39–41]. These concepts arose in the interviews and focus groups, with participants advocating for the PN acting as a bridge to adult providers and offering transition-related education. Having health care providers communicate directly with adolescents regarding self-management and transition planning was also highlighted as a key finding in the literature [39]. This was strongly supported by our study participants who acknowledged the importance of the PN role in offering developmentally-appropriate support. Interestingly, however, the literature suggests some young people report ambivalence about taking increased responsibility for their care [42] which suggests the PN account for the young person’s feelings about and readiness for transition in developing a tailored intervention.

Our study adds new information in several areas. These include tailoring support based on the unique needs of young people at the age of transition, and perspectives regarding duration of support post-transfer to adult care. Post-transfer involvement and follow-up with patients in the adult system is well supported in the literature, however, the duration of support and physical location of the PN widely varies among programs, and is poorly defined. A review by McBrien and colleagues (2018) [43] summarized 67 unique programs and showed that intervention frequency can be as little as 1 contact, with only 14 programs providing support for > 12 months. Many participants in this study thought that PN support should ideally continue as long as needed, but some acknowledged the practicality of resource limitations, citing the PN intervention needs to be time limited. These perspectives come from stakeholders who are intimately familiar with a pediatric patient population where patients have different levels of medical and psychosocial complexity, and also have various developmental stages which are often affected by the underlying disease process itself. The nature and duration of support needs to be individualized and patient-oriented, and flexible to accommodate diversity of patient and family needs.

This study found that the role of the navigator as a coach to help patients develop self-management skills is important. The shift in responsibility from parents (and pediatric health providers) to young people over time is well supported by existing models for young people with chronic health conditions, including the shared management model [44].

Participants in this study highlighted system navigation, specifically connection to services, bridging pediatric and adult systems and engaging primary care supports as a key role for the PN. There is substantial agreement in the literature about the need for PNs to help facilitate young people to access resources particularly in adult care, which tends to be larger and a more fragmented health system than pediatric care [2, 23, 45]. Despite common acknowledgement that all transitioning young people need a primary care provider, many of our participants noted there is a need for increased support and education of primary health care providers in caring for these patients. It is further recommended that training be provided to primary care providers to increase their understanding and management of young adults with complex needs and neurodevelopmental conditions [46]. However, there may be further unique barriers to caring for complex young people in a primary care setting which need to be explored [47, 48]. Very few of the transition interventions that have been evaluated involve a primary care component [49]. Given time constraints for primary care physicians, we acknowledge that having them play a key role in supporting connection to adult specialists may not be feasible [50–52]. Thus, involving a PN who acts as a liaison between primary and tertiary care is recommended based on our study findings.

## Strengths and limitations

A major strength is that our sample consists of perspectives of those in leadership, policy and executive roles within the transition field. This study has several limitations including a larger sub-group of participants from pediatric rather than adult settings, the absence of psychiatrists, and few participants working primarily in rural or large geographic regions. The participants recruited for this study were also limited to those involved in a provincial network tasked with the goal of examining best practices for children and adolescents with health conditions. Young people and their families were not part of the scope of this paper and we acknowledge the importance of youth and family perspectives in health care delivery. Input from these groups is extremely valuable and important in order to implement and maximize the effectiveness of patient navigation. Next steps of this project include a qualitative exploration of the needs and experiences of young people and caregivers in establishing best practices for a patient navigator intervention by way of individual interviews.

## Conclusions

This study explores how patient navigators can support young people with special health care needs and their families to successfully transfer from pediatric to adult care. The findings of this study will help guide the development of a transition navigator role in a diverse health system and will inform future interventional trials to evaluate the effectiveness of PN programs.

## Additional file

Additional file 1: Summary of interview guide. (DOCX 13 kb)

## Data Availability

The qualitative transcripts from this study will not be publicly shared in accordance with participant confidentiality.

## Abbreviations

PN: Patient navigator
SHCN: Special health care needs
