# Supplementary material for: Health care stakeholder perspectives regarding the role of a patient navigator during transition to adult care
Source: BMC Health Serv Res. 2019 Jun 17;19:390. doi: 10.1186/s12913-019-4227-6 (PMC6580652; doi:10.1186/s12913-019-4227-6)
Supplement: Supplementary file 1 — Summary of interview guide. (DOCX 13 kb) [file 12913_2019_4227_MOESM1_ESM.docx]

**Additional File 1: Summary of interview guide**

| **Question** |
| --- |
| ***Background****- to better understand participant roles, responsibilities and experiences working with young adults and their families* |
| What is your primary role? |
| What kinds of experience do you have working with youth, young adults and their families and in what context? |
| What roles have you played in youth transitioning to adult care? |
| What sorts of experiences have you seen people have during transition? |
| Have you ever wished you were able to do something different for these young people and their families? |
| ***Patient navigator existing knowledge****- to explore participants’ current perceptions and knowledge of patient navigator services* |
| Based on your current understanding, what is a patient navigator? |
| What are your opinions on the current navigators that exist? Is there one where you work? |
| In your experience, are the policies and procedures in place effective for youth? For their families? |
| What are unique issues in Alberta that impact the transition experience? |
| What are some policies/legislation that you think apply to patient navigators? |
| What barriers have you personally experienced when trying to support a youth and their family in transitioning to adult oriented services? |
| What are some of the ways in which the current policies for transition age youth are successful? |
| What do you think you would need to better support youth transitioning into adult oriented services? |
| ***Future development of patient navigator services****- to ascertain ideal roles and elements that key stakeholders feel should be included in the implementation of a patient navigator service* |
| Do you think the patient navigator should be located in the pediatric or adult system? Or both? |
| What should be the main role and background of the patient navigator? |
| Who do you think would benefit most? Who do you think would benefit the least? |
| What are the main gaps that his service would fill? |
| Would you suggest that the navigator work just with youth? What about their families? |
| What role does a navigator play on an interdisciplinary team? |
| What is the best method of communicating with youth and their families? |
| What are the main tasks, responsibilities and services of the patient navigator? |
| When would you suggest the patient navigator system start working with youth? |
| When would you suggest the patient navigator stop being offered to young people? |
| How will we know that a patient navigator has achieved its outcome? |
| How will we know it is working? |
| What system changes will we notice? |
| What changes will we notice in the youth? What about their families and communities? |
